# Retrospective analysis of the predictive factors of renal function loss after uninephrectomy in patients with chronic kidney disease G3 to G5

**DOI:** 10.1186/s40697-015-0089-y

**Published:** 2015-12-11

**Authors:** Dominique Dupuis, Georges Ouellet, Louise Roy

**Affiliations:** Department of Medicine, CHUM Hôpital Saint-Luc, 1058 Saint-Denis, Montréal, Québec H2X 3J4 Canada; Department of Medicine, Hôpital Maisonneuve-Rosemont, 5415, Boulevard de l’Assomption, Montréal, Québec H1T 2 M4 Canada

**Keywords:** CKD, Nephrectomy, Chronic renal failure, Nephropathy progression, Hyperfiltration

## Abstract

**Background:**

The rapid increase in glomerular filtration rate in a normal contralateral kidney after uninephrectomy is well known in living kidney donors but much less well described in chronic kidney disease (CKD). The purpose of this study is to determine the magnitude of this initial compensatory capacity in (CKD) groups 3 to 5 (G3 to G5) patients undergoing uninephrectomy and the clinical factors predicting it. This is a retrospective study of all cases (142) of uninephrectomy in patients with estimated glomerular filtration rate (eGFR; with MDRD equation) <60 ml/min/1.73 m^2^, between 2003 and 2010, in two University of Montreal-affiliated teaching hospitals.

**Methods:**

Baseline eGFR, patients’ comorbidities, and surgical characteristics and complications were noted. The change of eGFR after nephrectomy was evaluated; moreover, the expected post-op eGFR, i.e. without compensation by the contralateral kidney following surgery, was estimated in a sub-group of patients who had a preoperative renal scintigraphy and compared to the actual eGFR at hospital discharge.

**Results:**

The mean change of eGFR from baseline to hospital discharge was −5 ± 12 ml/min/1.73 m^2^ (−11 %; 95 % CI −16 to −6 %; *P* < 0.001). In univariate and multivariate analyses, baseline eGFR did not influence significantly these results. However, in the multivariate model, radical nephrectomy vs. partial nephrectomy and preoperative hypertension predicted a worse renal outcome. In the sub-group of patients with preoperative renal scintigraphy, the actual eGFR at hospital discharge was also higher than expected from the renal split function (13 ml/min/1.73 m^2^; 95 % CI 10 to 16; *P* < 0.001).

**Conclusions:**

After uninephrectomy, the contralateral kidney in patients with CKD G3 to G5 still has a clinically significant initial compensatory capacity. The compensation is statistically smaller if the patient had hypertension or a radical uninephrectomy. This initial compensation is rapid and most probably haemodynamic (hyperfiltration). However, most of the included patients had a CKD G3, limiting the strength of the conclusion for the G4 toG5 patients; the length of observation covers the early postoperative period, i.e. less than 2 weeks, in more than half of the cohort.

## Résumé

**Mise en contexte:**

L’accroissement rapide du débit de filtration glomérulaire du rein controlatéral à la suite d’une uninéphrectomie (uniNPX) est bien connu pour les donneurs de rein vivants, mais est beaucoup moins bien documenté dans le cas des patients souffrant d’une insuffisance rénale chronique (IRC).

**Objectif:**

L’étude avait pour objectif principal de déterminer à quel point la capacité de compensation initiale du rein controlatéral est maintenue chez les patients souffrant d’IRC de stade 3 à 5 après une NPX et quels facteurs cliniques sont susceptibles d’aider à prévoir la perte d’activité fonctionnelle du rein.

**Cadre et type d’étude:**

Il s’agit d’une étude rétrospective de 142 cas de NPX pratiquées entre 2003 et 2010 au sein de deux centres hospitaliers universitaires affiliés à l’Université de Montréal, sur des patients présentant un débit de filtration glomérulaire estimé (DFGe) par l’équation MDRD, inférieur à 60 ml/min/1.73 m^2^.

**Méthode:**

La mesure du débit de filtration glomérulaire préopératoire, les maladies concomitantes inscrites au dossier, les particularités de la chirurgie ainsi que les complications survenues par la suite, ont été notées pour chaque patient. La variation du DFG a été évaluée après la chirurgie. De plus, le DFG post-op attendu, calculé en ne tenant pas en compte la compensation offerte par le rein controlatéral après la chirurgie, a été estimé pour un sous-groupe de patients qui avaient subi une scintigraphie rénale préopératoire et comparé à la mesure du DFG faite au moment de la sortie de l’hôpital.

**Résultats:**

La variation moyenne du DFG entre les mesures prises avant la chirurgie et celles prises lors de la sortie de l’hôpital était de −5 ± 12 ml/min/1.73 m^2^ (−11 %; 95 % IC −16 % à −6 %; *P* < 0.001). Le DFG initial n’a en aucun cas influencé significativement les résultats qu’ils aient été analysés par un modèle d’analyse unidimensionnelle ou par un modèle d’analyse à variables multiples. Toutefois, dans le second type d’analyse, la présence d’hypertension préopératoire et le type de néphrectomie pratiquée (partielle ou radicale) ont laissé entrevoir de moins bons résultats. Enfin, le DFG mesuré à la sortie de l’hôpital chez les patients qui avaient subi une scintigraphie rénale préopératoire était plus élevé que le DFG prévu, calculé à partir de la division de la fonction rénale (13 ml/min/1.73 m^2^; 95 % IC 10 à 16; *P* < 0.001).

**Conclusions et limites de l’étude:**

À la suite d’une néphrectomie, le rein controlatéral des patients atteints d’insuffisance rénale chronique de stade 3 à 5 possède toujours une capacité compensatoire significative du point de vue clinique. Par contre, la compensation est moindre si le patient souffre d’hypertension avant la chirurgie ou lorsqu’il subit une uniNPX radicale, elle est également rapide et probablement hémodynamique (hyperfiltration). Il importe également de noter que la majorité des patients ayant participé à l’étude avait une IRC de stade 3 et que ceci ne permet pas de tirer des conclusions sans équivoque pour les patients de stades 4 et 5. De plus, les observations se sont déroulées sur une très courte période, moins de deux semaines pour plus de la moitié de la cohorte, immédiatement après la chirurgie.

## What was known before

The course of kidney function changes after nephrectomy has been well described in living kidney donors. Data on outcomes in patients with glomerular filtration rate less than 60 mL/min are limited, however.

## What this adds

Loss of kidney function in the immediate postoperative period was low in proportion to the renal tissue removed, suggesting the occurrence of hyperfiltration. This information is useful for preoperative counselling in this population.

## Background

The rapid increase in glomerular filtration rate (GFR) in a normal contralateral kidney after uninephrectomy (compensation) is established by studies on living kidney donors [1–4]. A rapid initial increase in GFR up to 60–70 % of pre-donation GFR value is observed as early as the first week post-nephrectomy [2, 4] and suggests a haemodynamic response. This is followed by hypertrophic changes in the subsequent weeks to months with a gradual increase and then a stabilisation of kidney function for up to 20 years [5]. Among living kidney donors, favourable long-term outcomes have usually been reported; recent data suggests higher risk of end-stage renal disease (ESRD) when compared to healthy controls, although the absolute risk remains small [6, 7]. However, patients undergoing uninephrectomy for renal cell carcinoma differ from this carefully selected healthy living donor population: they have comorbidities and may have significant chronic kidney disease (CKD) prior to surgery [8]. Therefore, the observed renal compensation and relative benign course observed in living donors might be different in those patients, especially those with prior CKD.

Recently, several studies have evaluated the kidney and global outcomes of patients undergoing uninephrectomy [9–11]. Many have done so focusing on different surgical techniques that could preserve renal function, mainly partial nephrectomy [12–14]. Age, lower preoperative estimated glomerular filtration rate (eGFR), hypertension, proteinuria, postoperative acute kidney injury (AKI), tumour size, ischemia time and radical nephrectomy have been reported as predictive factors of poorer postoperative renal function [15–19]. Only few of these studies have specifically included patients with prior advanced CKD [15, 16, 20]. In a model to identify predictive factors of lower postoperative renal function, Lane BR et al. showed less than 10 % difference between preoperative eGFR and ultimate eGFR in 290 patients with CKD groups 3 to 5 (G3 to G5) over a 1.5-year follow-up after partial nephrectomy [16]. Takagi T et al. also observed that after partial nephrectomy, most patients with CKD G3 and G4 keep a stable renal function; they also showed in a small group of radical nephrectomy (51 patients, 25 % G4 and 75 % G3) that eGFR is rather stable in the first following year [15, 20]. It suggests that, even in patients with advanced renal disease, the remaining renal parenchyma maintains compensatory capacity.

Counselling patients regarding the potential worsening of their kidney function following radical nephrectomy requires more knowledge concerning this specific population. The purpose of this study was to determine if the initial compensatory capacity of the contralateral kidney is still present in our population of CKD G3 to G5 patients after radical nephrectomy. The secondary objective was to determine which factors influence this compensatory capacity.

## Methods

### Study population

We retrieved the cases of uninephrectomy between January 1, 2003, and December 31, 2010, in Hôpital Maisonneuve-Rosemont and the Centre Hospitalier Universitaire de Montréal (CHUM), two university-affiliated hospitals in Montreal. Among those cases, we selected the patients who had a baseline eGFR ≤60 ml/min/1.73 m^2^. Patients with metastatic renal cancer, patients with transplants and patients already on dialysis were excluded. Baseline eGFR; patients’ comorbidities; surgical characteristics; and complications, medication and hospital length of stay were noted, according to the medical record available. Baseline eGFR was calculated according to the four-variable Modification of Diet in Renal Disease (MDRD) study equation, with the last available preoperative creatinine. Postoperative eGFR is defined as calculated at discharge from hospital. Data on serum creatinine at hospital discharge and 6 to 12 months postoperatively (if available) were collected.

### Statistical analysis

Categorical variables are presented as percentages and continuous variables as means and standard deviations (SD).

We compared the preoperative and postoperative eGFR and calculated the proportional change. Moreover, for patients who had a preoperative renal scintigraphy, we estimated the expected postoperative eGFR according to split renal function (“expected-corrected”) and then compared this expected-corrected eGFR to the actually observed postoperative eGFR, to see if it would refine our prediction of postoperative kidney function. We used paired *t* tests to evaluate the variation between preoperative and postoperative eGFR and between observed and expected postoperative eGFR (and expected-corrected for the scintigraphy sub-group). Univariate analyses were repeated in significant subsets of patients: radical or partial nephrectomy; patients with and without scintigraphy; and patients with baseline eGFR ≤45 or 45 to 59 ml/min/1.73 m^2^. *P* values of less than 0.05 were considered statistically significant.

Finally, we used a multivariate linear regression model including age, hypertension, proteinuria, baseline eGFR, length of hospital stay, complications in the first week post-surgery, radical nephrectomy and preoperative cessation of angiotensin-converting enzyme inhibitor (ACEI) or angiotensin receptor blocker (ARB). It allowed us to evaluate the impact of those factors on the variation of eGFR after nephrectomy (%) and the difference between actual and expected-corrected eGFR for patients with scintigraphy. *R* squared and ANOVA tests were used for analyses of the linear regression models. *P* values of less than 0.05 were considered to indicate statistical significance in those models as well.

IBM SPSS Statistics 21 was used for statistical analyses.

## Results

Between January 1, 2003, and December 31, 2010, 847 nephrectomies were performed in these two hospitals. Of these nephrectomies, 183 were performed in patients with pre-op eGFR ≤60 ml/min/1.73 m^2^, who were not on dialysis and did not have a renal graft in the past; 142 patients were included in the study. Excluded patients had advanced metastatic cancer or incomplete medical record. Patients’ baseline characteristics are presented in Table 1. Diabetes, hypertension, proteinuria and the use of an ACEI or ARB prior to surgery were, as expected, more prevalent among patients with eGFR <45 ml/min/1.73 m^2^. The majority of our patients (82 %) underwent a radical nephrectomy; the remainder underwent partial nephrectomy. There was an equal proportion of partial vs. radical nephrectomy in the group of patients with eGFR < or ≥45 ml/min/1.73 m^2^, and whether or not a preoperative renal scintigraphy was performed. The pathology was renal cell carcinoma (43 %), urothelial carcinoma (21 %), benign tumour (19 %) and other causes (17 %).

**Table 1:** Baseline characteristics

	Baseline eGFR (ml/min/1.73 m^2^)		
	<45	45 to 59	Total
Characteristics	(*n* = 42)	(*n* = 100)	(*n* = 142)
eGFR (ml/min/1.73 m^2^)	35 ± 8	53 ± 4	48 ± 10
Age (years)	70 ± 9	68 ± 10	69 ± 10
Male	28 (67)	52 (52)	80 (56)
Diabetes	23 (55)	26 (26)	49 (35)
Hypertension	39 (93)	71 (71)	110 (78)
Proteinuria	22 (54)	29 (29)	51 (36)
Vascular/heart diseases	16 (38)	40 (40)	56 (39)
Preoperative ACEI or ARB	25 (60)	49 (49)	74 (52)
Radical nephrectomy	34 (81)	82 (82)	116 (82)
Hospital stay length (days)	12 ± 18	8 ± 7	9 ± 12
Complications at 1 week postoperatively^a^	16 (38)	45 (45)	61 (43)
Hypotension	8 (19)	20 (20)	28 (20)
Infectious complications	5 (12)	11 (11)	16 (11)
Cardiovascular complications	10 (24)	8 (8)	18 (13)

Data presented as mean ± standard deviation for continuous variables and number of cases (%) for dichotomous variables

*eGFR* estimated glomerular filtration rate by Modification of Diet in Renal Disease study equation, *ACEI* angiotensin-converting enzyme inhibitor, *ARB* angiotensin II receptor blocker

^a^Types of complications are reported for more information, but for analysis purposes, patients were classified as having any complication or no complication. One patient could have one or more types of complications; the (%) represents the proportion of total cohort (142) for each type of complication

Preoperative imaging reports describing the non-affected kidney were available in half of the patients, but renal size and volume were not systematically assessed and therefore could not be used as a means of estimating the renal function. However, preoperative renal scintigraphy was available in 42 % of the patients; in these patients, the mean proportional function of the kidney to be removed was 40 ± 15 %, and their baseline eGFR tended to be lower than for patients for whom a preoperative scintigraphy was not performed (44 ± 10 vs. 51 ± 7 ml/min/1.73 m^2^). Imaging of the contralateral kidney (hyperechogenicity or atrophy) was described as abnormal in 13 % of patients without scintigraphy and 6 % of patients with scintigraphy.

The mean duration of hospital stay was 9 ± 12 days with a median of 6 days; 43 % of hospitalisations were complicated by hypotension, infectious event or cardiovascular event in the first week. The majority (88 %) of patients who stayed hospitalised more than 14 days had complications in the first week. There was no association between a longer hospital stay and eGFR at discharge. Among patients with available follow-up data at 1 year (*n* = 82), 6 patients were on dialysis and 7 had died.

**Fig. 1:**
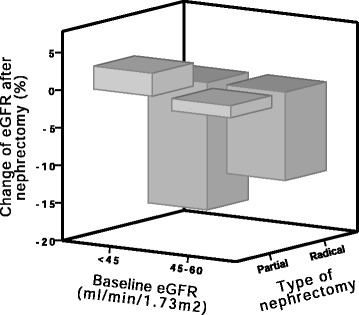
*Change of eGFR after nephrectomy* partial vs. radical. *Change of eGFR* represents the difference between eGFR at hospital discharge and eGFR at baseline in proportion to baseline eGFR (%)

The eGFR decreased significantly after nephrectomy; the mean difference from baseline to hospital discharge was −5 ± 12 ml/min/1.73 m^2^ or −11 % (95 % CI −16 to −6 %; *P* < 0.001), which is less than the theoretically expected 50 % without any compensation from the remaining kidney. Seventy-four patients were using ACEI or ARB prior to hospitalisation; the medication was discontinued before surgery in 29 of them; the mean decrease in eGFR was not significantly different whether the ACEI or ARB were discontinued (−4 ± 12 ml/min/1.73 m^2^ or −7 ± 33 %) or not (−5 ± 12 ml/min/1.73 m^2^ or −11 ± 27 %). The decrease in eGFR was significantly steeper in patients who underwent radical nephrectomy −6 ± 12 ml/min/1.73 m^2^ or −13 ± 29 % vs. partial nephrectomy −0.2 ± 10 ml/min/1.73 m^2^ or −0.2 ± 21 % (*P* = 0.03) (Fig. 1). However, the decrease in eGFR was not significantly different according to baseline eGFR: −3 ± 11 ml/min/1.73 m^2^ or −13 ± 37 % for patients with baseline eGFR <45 ml/min/1.73 m^2^, vs. −5 ± 13 ml/min/1.73 m^2^ or −10 ± 24 % for patients with baseline eGFR 45–60 ml/min/1.73 m^2^. There was a non-significant small increase in eGFR in patients with baseline eGFR <45 ml/min/1.73 m^2^ after partial nephrectomy.

According to the multivariate model, patients who had preoperative hypertension and patients who underwent radical nephrectomy had a significantly more important proportional decrease in their eGFR (decrease of eGFR/baseline eGFR in %) at hospital discharge. However, a lower baseline eGFR in this multivariate model did not predict a more significant decrease in eGFR at hospital discharge.

**Fig. 2:**
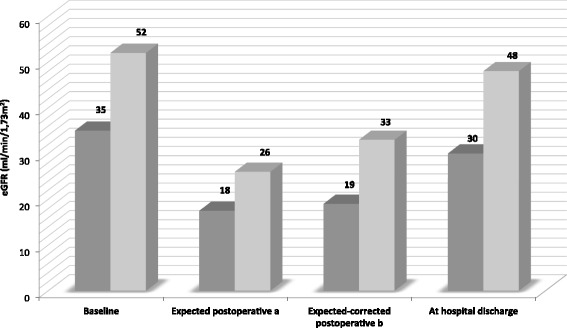
Mean eGFR according to baseline in patients with preoperative renal scintigraphy (*n* = 59). Baseline eGFR <45 ml/min/1.73 m^2^. Baseline eGFR 45 to 59 ml/min/1.73 m^2^. a *Expected postoperative* if 50 % decrease from baseline eGFR. b *Expected-corrected postoperative* if loss of function is determined by preoperative renal scintigraphy

In the sub-group of patients who had a preoperative scintigraphy, the mean change of eGFR was −4 ± 12 ml/min/1.73 m^2^ or −10 ± 27 %. The actual eGFR at discharge was higher than the eGFR predicted according to renal split function on scintigraphy (here called “expected-corrected”); the difference between actual and expected-corrected eGFR was 13 ml/min/1.73 m^2^ (95 % CI 10 to 16; *P* < 0.001) (Fig. 2). The scintigraphy only slightly changed the results from an expected eGFR using the theoretical 50 % decrease, as the mean split function of the nephrectomised kidney on scintigraphy was 40 % (Fig. 2). Using the difference between actual and expected-corrected postoperative eGFR as the dependant variable, the multivariate model here shows that, among patients with a preoperative scintigraphy, radical nephrectomy also predicts a significantly worse renal outcome (*P* = 0.001); meaning that the difference between actual and expected eGFR was smaller in those who underwent a radical nephrectomy. We also observed that in this sub-group of patients with scintigraphy, a longer hospital stay was associated with a significantly higher eGFR than predicted at hospital leave, in other words, patients who stayed in hospital the longest had a higher eGFR at hospital leave than those with a shorter hospital stay.

Among the 8 patients with CKD G4, the mean actual decrease in renal function after radical uninephrectomy was −4 ± 13 ml/min/1.73 m^2^ or −25 ± 56 %. Two CKD G4 patients started dialysis within a week postoperatively. The only patient with CKD G5 was dialysed 2 days after surgery. The three patients dialysed in the first week after surgery had a preoperative eGFR ≤20 ml/min/1.73 m^2^.

Data on eGFR 6–12 months post-nephrectomy were available in 74 patients; their eGFR decreased by a further 4 ± 8 ml/min/1.73 m^2^ after hospital discharge.

## Discussion

Rapid partial correction of GFR is well described in healthy living kidney donors [1–4]. A similar observation has been done in patients after partial nephrectomy. However, very few data are available after radical nephrectomy. Our retrospective study indicates that kidneys preserve a capacity of compensating a loss of parenchyma, even in CKD patients. This study helps us in answering a pragmatic question for patients with CKD who face the necessity of undergoing a radical uninephrectomy. In our population, the mean actual loss of renal function at hospital discharge was only 11 %, much less than the theoretical 50 %.

Patients with a planned nephrectomy and CKD are referred to the nephrology clinic as part of their preoperative work-up, in order to assess and discuss the loss of renal function and potential risk of starting haemodialysis. Considering our data, we can improve our counselling and decision-making regarding the risk and early management of chronic renal failure following nephrectomy. A new baseline postoperative eGFR is reached as quickly as 4 days post-nephrectomy [16], and the eGFR reached in the first few months is sustained in time over a year [21], which made us comfortable in using MDRD for GFR estimation at hospital discharge, on average 9 days after nephrectomy.

Twenty-nine of our patients had an ACEI or ARB stopped prior to surgery, but the subsequent expected better outcome in eGFR was not statistically significant, when compared to patients who underwent surgery still on ACEI or ARB; this could be due to lack of power, since these patients represented only 20 % of our study group.

A renal scintigraphy was available for 42 % of our patients. It has the theoretical benefit of better approximating the expected decrease in eGFR after nephrectomy; however, our data do not support this advantage if prescribed routinely pre-nephrectomy, as shown in Fig. 2. It is still possible that in patients with a marked asymmetry of GFR between kidneys, scintigraphy might be helpful in predicting the outcome; volume estimation methods by magnetic resonance or ultrasound could also permit an estimation of the differential function but were not available for this retrospective report. The multivariate model in patients who underwent renal scintigraphy preoperatively showed an even higher eGFR than expected in those patients with a longer hospital stay; this could suggest a continued increase in GFR post-op, as described in some studies on living donors, an increased total body water because of hydration during hospitalisation or a decrease in muscular mass in patients submitted to a long hospital stay.

In recent years, partial nephrectomy has been advocated as the method of choice to reduce renal function loss after nephrectomy. The 2010 update of the European Association of Urology Guidelines on Renal Cell Carcinoma designates indications of partial nephrectomy (nephron-sparing nephrectomy); they establish that having a contralateral kidney affected by a condition that could impair renal function in the future is a relative indication [22]. Nowadays, we might expect an even better outcome of postoperative eGFR with the more systematic use of partial nephrectomy. This is suggested by a better renal outcome in our patients with partial nephrectomy and is in accordance with previous reports describing less ESRD and CKD with partial nephrectomy [19], although some data question the positive impact of partial nephrectomy in CKD G3 [20, 23].

There are several limitations in our study. First, this is a retrospective study, with biases of selection and uncontrolled confounding factors. Second, the limited data at 6 to 12 months shows a small further decrease (4 ml/min/1.73 m^2^) in GFR during the first year following nephrectomy, but the course of renal function in the year after nephrectomy is not clearly described by our data. With lower GFR from any cause comes hyperfiltration that leads to further gradual decreasing in GFR; this has been observed also in post-nephrectomy [17]. The progression of CKD and the risk of ESRD, especially after 1-year post-nephrectomy, are not addressed by this study. Another limiting factor for the interpretation of the results is the small number of patients who had an eGFR lower than 30 ml/min/1.73 m^2^, limiting our conclusions mainly to CKD G3 patients; only 8 patients had CKD G4 and one had CKD G5.

## Conclusions

After total uninephrectomy, the contralateral kidney in patients with CKD G3 and possibly G4 still has a clinically significant initial compensatory capacity. The preservation of renal function is smaller if the patient has preoperative hypertension or undergoes a radical nephrectomy. This compensation seems rapid and hence is most probably haemodynamic, as described in living kidney donor models. Further research is needed to confirm these findings in a prospective manner and to further evaluate the outcome of CKD G5 patients, as well as the long-term renal outcome.
